# *Dermatophilus congolensis* infection in sheep and goats in Delta region of Tamil Nadu

**DOI:** 10.14202/vetworld.2017.1314-1318

**Published:** 2017-11-08

**Authors:** M. Ananda Chitra, K. Jayalakshmi, P. Ponnusamy, R. Manickam, B. S. M. Ronald

**Affiliations:** 1 Department of Veterinary Microbiology, Veterinary College and Research Institute, Tamil Nadu Veterinary and Animal Sciences University, Orathanadu - 614 625, Thanjavur, Tamil Nadu, India; 2 Department of Veterinary Medicine, Veterinary College and Research Institute, Tamil Nadu Veterinary and Animal Sciences University, Orathanadu - 614 625, Thanjavur, Tamil Nadu, India

**Keywords:** 16S rRNA sequence analysis, *Dermatophilus congolensis*, sheep and goats, Tamil Nadu

## Abstract

**Aim:**

The study was conducted to isolate and identify *Dermatophilus congolensis* (DC) using conventional and molecular diagnostic techniques in scab materials collected from skin infections of sheep and goats in the Delta region of Tamil Nadu.

**Materials and Methods:**

A total of 20 scab samples collected from 18 goats and 2 sheep from Nagapattinam, Thanjavur, and Tiruvarur districts of Tamil Nadu. Smears were made from softened scab materials and stained by either Gram’s or Giemsa staining. Isolation was attempted on blood agar plates, and colonies were stained by Gram’s staining for morphological identification. Identification was also done by biochemical tests and confirmed by 16S rRNA polymerase chain reaction (PCR), followed by sequencing and phylogenetic analysis of the amplified product.

**Results:**

The peculiar laddering arrangement of coccoid forms in stained smears prepared from scab materials revealed the presence of DC. Isolated colonies from scab materials of sheep and goats on bovine blood agar plate were small, hemolytic, rough, adherent, and bright orange-yellow in color, but some colonies were white to cream color. Gram-staining of cultured organisms revealed Gram-positive branching filaments with various disintegration stages of organisms. 16S rRNA PCR yielded 500 bp amplicon specific for DC. Sequence analysis of a sheep DC isolate showed 99-100% sequence homology with other DC isolates available in NCBI database, and phylogenetic tree showed a close cluster with DC isolates of Congo, Nigeria, and Angola of Africa. Genes for virulence factors such as serine protease and alkaline ceramidase could not be detected by PCR in any of the DC strains isolated of this study.

**Conclusion:**

The presence of dermatophilosis in Tamil Nadu was established from this study.

## Introduction

Dermatophilosis is an exudative pustular dermatitis that affects many animals, and occasionally, humans. Dermatophilosis is caused by the bacterium *Dermatophilus congolensis* (DC). The clinical appearance and parts of the body affected are varied in different hosts depending on their nutritional and immune status, intense rainfall, and mechanical trauma. The infection may give rise to the formation of dense scabs on the skin or moist lesions with thickened, folded skin, especially in areas of the perineum in ruminants and pastern in horses [1]. When lesions are exposed to prolonged wetting, with or without secondary infection, exudative lesions may be present.

The disease causes considerable economic loss as a result of lowered production, increased culling, downgrading of hide, and death [2]. There is no single treatment specific for dermatophilosis.

This study was conducted to isolate and identify *Dermatophilus congolensis* (DC) using conventional and molecular diagnostic techniques in scab materials collected from skin infections of sheep and goats in the Delta region of Tamil Nadu.

## Materials and Methods

### Ethical approval

As we had collected scabs and swabs from infected animals for routine clinical diagnostic procedures, no ethical committee approval was obtained for this study. However, we obtained informed consent from all the owners involved in this study for sample collection and we maintained the confidentiality of the diagnostic results.

#### Clinical specimen collection

**Figure-1:**
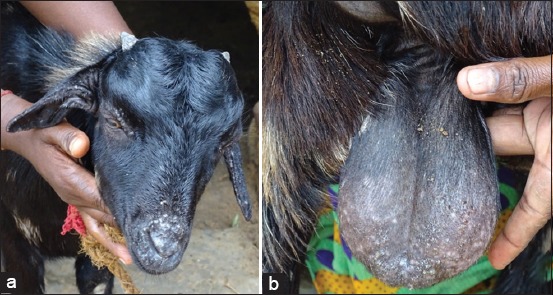
Dermatitis with dried scabs on the face, ears (a) and scrotum (b) of a goat with dermatophilosis.

A total of 20 samples comprising 18 samples from non-descriptive goats and 2 samples from sheep with clinical signs of matted hairs and thick scab on the face, ear, udder, or scrotal area (Figure-1) and on the dorsal area of the body were collected during October 2015 to February 2016 from Nagapattinam, Tiruvarur, and Thanjavur districts of Tamil Nadu, India. Only small thick dried scabs were seen on some animals which were on the recovering phase of the disease. Sterile skin scabs and skin swabs from lesions were collected for laboratory examination.

### Direct microscopical observation

The aseptically collected scabs were crushed and softened in the water and made smear on a clean oil-free microscopical slide. The air-dried and heat-fixed smear was then stained with Gram-stain and/or Giemsa stain and seen under oil immersion objective of the microscope.

### Isolation

Isolation was attempted using Haalstra’s method [3] based on the nature of capnophilic motile zoospore of DC. The loopful of sample was inoculated on the blood agar plates by quadrangle streaking method and incubated at 37°C in a candle jar for 48 h. Individual colonies were stained and examined for DC. The preliminarily identified organisms were subjected to further biochemical tests and for polymerase chain reaction (PCR).

### DC identification using 16S rRNA gene sequencing and virulence genes detection by PCR

DNA extraction from scab material was performed using tissue DNA extraction spin column kit (Qiagen, Germany) by following the manufacturer’s instruction. DNA from isolated bacteria was extracted by boiling method [4]. PCR was carried out using the already published primers (Table-1) targeting DC 16S rRNA by Shaibu *et al*. [5] for species identification and alkaline ceramidase by Garcia-Sanchez *et al*. [6] and serine protease by Garcia-Sanchez *et al*. [7] for virulence genes detection. PCR was performed in a reaction volume of 10 µl containing approximately 100 ng of genomic DNA, 5 pmol of each primer, and 2× master mix (Ampliqon, Denmark). Cycling conditions were 94°C for 3 min, followed by 30 cycles of denaturation at 94°C for 30 s, annealing at 55°C for 30 s, extension at 72°C for 30 s, and a final extension cycle of 5 min at 72°C. PCR products were loaded on a 1.5% agarose gel for electrophoresis, visualized with ethidium bromide, and documented.

**Table-1:** Primers used in this study along with amplicon size and annealing temperature.

Primer name	Sequences	Ampliconsize (bp)	Annealing temperature
16S rRNA	F5’ACATGCAAGTCGAACGATGA3’ R5’ACGCTCGCACCCTACGTATT3’	500	55
DC-SP	F5’GATGGAAAATGCAAGGAGCAG3’ R5’GTCTTCGGGGTCCATGAACAT3’	600	55
DC-AC	F5’CTTCAGCAGAAAATTCACCA3’ R5’CGTACATTCCCGGAATCTTC3’	430	55

### DNA sequence analysis

Purification of the amplicon was done using PCR purification kit (Real Biotech Corporation, Taiwan) as per the manufacturer’s instruction. DNA sequencing of purified nucleotides was done with an automated high throughput nucleic acid sequence of Applied Biosystems 3500 Instrument model. Homology searches were performed with the NCBI database and BLAST. Alignment and phylogenetic tree analysis were carried out using the Mega5.2 software.

### Nucleotide sequence accession number

The GenBank accession number for the partial 16S rRNA nucleotide sequence of DC strain isolated from sheep is KX812810.

## Results and Discussion

Dermatophilosis may occur in acute, subacute, chronic, and latent forms, either in a generalized form or as localized lesions in different body sites, such as the dorsal region, the feet, the external genital area, mammary skin, and the head area. In our study, we have generally seen the subacute dermatophilosis in sheep and goat without any mortality. The affected animals had decreased weight gain, and infected young ones of less than 1 month showed stunted growth compared to uninfected ones in the same village. Only animals in close contact with the infected animals were affected within the village, and many animals recovered with little intervention of veterinarians. In some villages, the owners of affected animals had applied the mixture of neem oil and turmeric powder over the lesion resulted in the healing.

The first case of dermatophilosis was recorded in cattle in Belgian Congo in 1915 [8] with the name dermatose contagieuse (*Impetigo contagieux*). In India, it was first described in a buffalo calf in 1976 [9]. Pal [10] reported dermatophilosis in buffalo, cattle, goat, antelope, horse, and human from North and Western parts of India. In recent years, dermatophilosis in cattle and buffalo was reported in Kerala [11] and Andhra Pradesh [12]. In Kerala, a particular type of dermatitis of the lower limbs of cattle known as pododermatitis, characterized by the formation of thick scabs and crusts with cracks and fissures, was reported [11]. There is no previous report of dermatophilosis in animals in Tamil Nadu.

The hot and humid climate, persistently moist conditions, the presence of biting flies, ticks, and immunosuppression due to the stress associated with pregnancy and lactation were implicated as the predisposing factors for the increased incidence of dermatophilosis among dairy cattle in Kerala [13]. In the present study, the affected animals were not infested with ticks, but it was the rainy season and animals were exposed to rain. This may be the predisposing factor for the incidence of dermatophilosis in the Delta region of Tamil Nadu.

**Figure-2:**
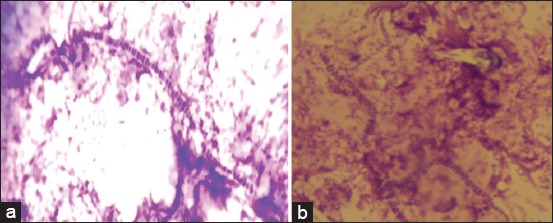
Long filaments with multiple rows of cocci (tram-track appearance) seen in scab materials stained with Gram’s (a) and Giemsa staining methods (b).

Stained smears from scab materials revealed branching filaments with cocci arranged in vertical and horizontal rows (Figure-2a) which are the characteristic of DC made our diagnosis easier and earlier. Smears of dried scab materials collected from recovering animals revealed very few tram track appearance with more of ovoid or spherical cocci. The distinctive pattern was visible more vividly with less background color in Giemsa-stained (Figure-2b) than in Gram-stained smears as suggested in many literature [1,13]. DC generally infects epidermis layer only and occasionally infects the dermis. Scabs characteristically comprise alternating layers of parakeratotic keratinocytes invaded with branching bacterial filaments and infiltrates of neutrophils in serous exudate. This gives a palisaded appearance in stained sections [1].

**Figure-3:**
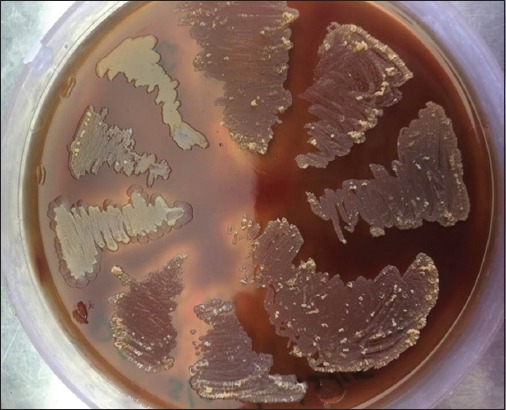
Colonies of *Dermatophilus congolensis* isolates on blood agar show hemolysis and varied colony morphology.

**Figure-4:**
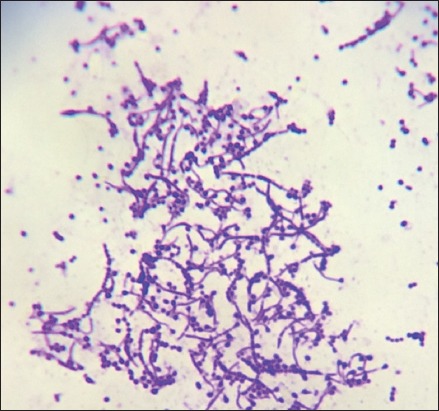
Gram’s staining of a colony shows fragmentation of branching filaments into cocci.

Isolated colonies from scab materials of sheep and goats on bovine blood agar plate were small, rough, adherent colonies of bright orange-yellow color, but some colonies were white to cream color and all isolates caused complete hemolysis within 48 h (Figure-3). Gram-staining of cultured organisms revealed Gram-positive branching filaments with various disintegration stages of organisms (Figure-4). Colonies of sheep and goat scab materials initially showed more branching filaments with moderate coccoid forms and pitting into agar surface. On repeated subculture of the isolated colonies resulted in the white, less adherent colonies with few branching filaments and more of small coccoid organisms. Variation in the shape, color, and texture of the colonies of different isolates and even of the same isolate on the same agar plate was also reported earlier [13,14]. It has been observed that continued growth in air stimulates the production of the cocci, which are commonly yellow in color.

The observation of distinctive ladder pattern of DC in stained smears is an effective and low-cost method of diagnosis which can easily be adapted in basic diagnostic laboratory available in the nearby areas. However, in scab materials collected from chronically infected animals or secondarily infected cases or recovering animals or wet scabs, only free cocci may be present which necessitate the reliable and sensitive advanced technique such as immunofluorescent test and PCR [1].

**Figure-5:**
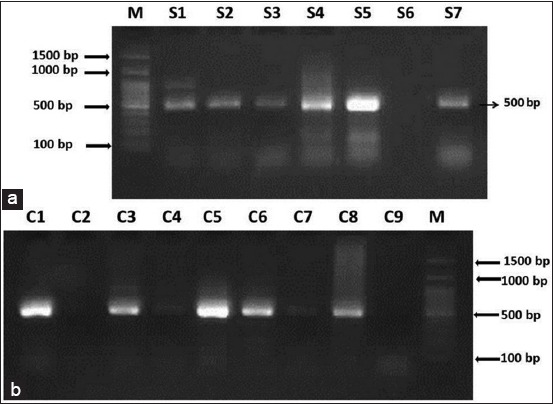
(a-b) Agarose gel electrophoresis showing the polymerase chain reaction amplified product of 500 bp for 16S rRNA gene of *Dermatophilus congolensis*. M: 100 bp DNA ladder; S1-S7: Scab materials; C1-C9: Colonies from blood agar plates.

The 16S rRNA PCR resulted in amplification of 500bp segment from all collected scab materials and suspected DC culture as shown in Figure-5a and b, respectively. No band was observed with negative control. The results obtained from the amplification of a 500bp segment of the 16S rRNA of DC agree with the finding of Wen-Xing *et al*. [15], Shaibu *et al*. [5], and Oladunni *et al*. [16]. The 16S ribosomal RNA gene has been found useful in the diagnosis of many bacterial organisms because of the highly conserved nature of this gene in most of the bacterial organisms. These pairs of primers were found to be highly specific in detecting DC by discriminating from other bacteria, and this PCR was recommended as a good diagnostic technique for DC isolates from cattle, sheep, and goat [5]. Direct amplification of 16S rRNA gene from scab samples is a useful tool in the diagnosis of DC infection, especially in cases of recovering animals, chronically infected animals or wet scab where the organisms are generally exist as scattered cocci rather than a distinguished tram-track pattern. This will make the diagnosis easy and also gives a confirmatory diagnosis.

**Figure-6:**
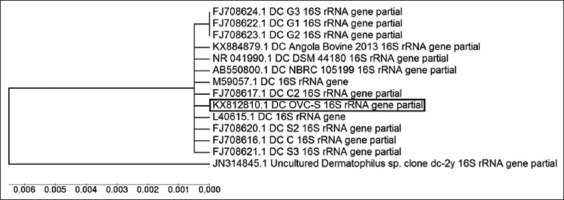
Phylogenetic tree based on partial 16S rRNA gene sequences of *Dermatophilus congolensis* isolate.

Sequence analysis of a sheep DC isolate showed 99-100% sequence homology with other DC isolates available in NCBI database, and phylogenetic tree showed a close cluster with DC isolates of Congo, Nigeria, and Angola of Africa (Figure-6). This is the first DC isolate 16S rRNA gene sequences of Asian origin submitted to NCBI as there is no DC 16S rRNA sequence of Indian or Asian isolates available in NCBI database for comparison and for phylogenetic tree analysis.

Even though the pathogenesis of dermatophilosis is not well understood, it is reported that DC is hemolytic [17] and produces phospholipases [18], proteolytic enzymes [19,20], alkaline ceramidase [6], and serine protease [7]. Many of these virulence factors were not characterized widely in DC isolates of the different geographical area, but these studies reported a high degree of phenotypic interstrain variation between DC strains.

There is no amplification of alkaline ceramidase and serine protease genes in PCR detected in our DC isolates. The absence of amplification for these genes may either due to the absence of genes or changes in the nucleotide sequence at the primer binding sites in these Indian isolates. Garcia-Sanchez *et al*. [6], who sequenced the alkaline ceramidase genes of DC, reported that the gene was present in all their DC isolates. They had also observed that there was a minor second band amplified in ovine DC isolates in addition to the major band of 430 bp. We could identify the serine protease gene sequences but not the alkaline ceramidase gene sequence in the shotgun whole genome of the DC strain DSM 44180.

## Conclusion

The DC infection in India is underdiagnosed due to lack of awareness among veterinarians as well as physician. This resulted in the absence of a report of DC in Northern India in the recent years. Importance of this disease is also undermined due to self-limiting and seasonal occurrence nature of this disease in many incidences and also due to the amenability of these organisms to many antimicrobials.

## Authors’ Contributions

MAC has designed and carried out the laboratory work, analyzed and compiled results, and also prepared the manuscript. KJ, PP, RM, and BSMR have equally contributed in the collection and processing of samples and in final editing. All authors read and approved the final manuscript.
